# Role of VEGF-A in chronic pain

**DOI:** 10.18632/oncotarget.14615

**Published:** 2017-01-13

**Authors:** Richard P. Hulse

**Affiliations:** Cancer Biology, School of Medicine, University of Nottingham, Queen's Medical Centre, Nottingham, UK

**Keywords:** degeneration, neuropathy, VEGF-A, nociceptor, pain, Neuroscience

Chronic pain is highly debilitating and impacts greatly upon the patients' wellbeing and quality of life. Chronic pain arises due to disease or treatment that cause dysfunction to the sensory nervous system, including arthritis, diabetes and chemotherapy induced sensory neuropathy. In diabetic patients, for example, sensory neurons in the peripheral and central (spinal cord) nervous system become hyperactive. This is replicated in rodent models of neuropathic pain, with C fibre and A fibre nociceptors responding with an increase in neuronal evoked and ongoing activity [1] as well as the onset of central sensitisation in the spinal cord. However, effective treatment for chronic pain is still a huge unmet clinical need.

Development of neuropathic pain is modulated by changes in expression and/or activity of voltage gated ion channels, neuropeptides and growth factors (such as upregulation of nerve growth factor in cancer and arthritic pain), which can influence sensory neuron excitability as well as altered structural components (sensory nerve fibre aberrant growth or degeneration) of the sensory neuron. Recent evidence now presents a prominent role for the angiogenic growth factor; vascular endothelial growth factor-A (VEGF-A) to impact upon the sensory nervous system, with nociceptive and neuroprotective roles acknowledged [2, 3] .

VEGF-A is widely recognised to regulate blood vessel function and integrity. However, alterations in VEGF-A expression and signalling profiles is strongly associated with a number of angiogenic related pathologies such as diabetes, arthritis and cancer, all of which are associated with chronic pain development. VEGF-A acts upon the sensory neuron by inducing sensory neuron axonal outgrowth and survival [2, 4, 5], and in early rodent studies highlighting a role of VEGF-A in inflammatory pain. The VEGF-A family consists of a number of differing isoform subtypes, with alternative splicing of exon 8 giving rise to VEGF-A_xxx_a (proximal splice site selection) and VEGF-A_xxx_b (exon 8b distal splice site selection) [6] . Differing exon content gives each of these isoforms distinct signalling and unique functional attributes compared to the sister isoforms [2, 4]. Therefore understanding the balance of these pro and anti-nociceptive VEGF-A isoforms and the control of exon 8 splice site selection is crucial in the development of analgesics.

The VEGF-A family signal through receptor tyrosine kinases VEGF R1 and VEGF R2 in addition to neuropilin co-receptors. VEGFR2 is expressed on myelinated A fibre and unmyelinated C fibre sensory neurons and is activated in rodent models of neuropathic pain. Such activity is associated with increased VEGF-A_xxx_a and reduced VEGF-A_xxx_b expression in both the peripheral (DRG, skin) [7] and central (spinal cord) [3] sensory nervous systems. Administration of VEGF_xxx_a sensitises C fibre mechanically sensitive nociceptors to which leads to increased transmission of pain signals and development of mechanical allodynia [7]. This is mediated by transient receptor potential vanilloid 1 (TRPV1) and TRPA1 channel activity. VEGF-A_165_a potentiates capsaicin (TRPV1 agonist) induced DRG sensory neuronal responses and TRPV1 phosphorylation. Consequently, VEGF-A_165_a mediated mechanical hyperalgesia was completely abolished following ablation of TRPV1 signalling either through TRPV1 knockout mice or pharmacological inhibition [7]. It is important to note that the anti-angiogenic VEGF-A_165_b suppresses TRPV1 and TRPA1 mediated activation of DRG sensory neurons, C fibre sensitisation and neuropathic pain [7], independent of VEGF-A_165_a induced activity, therefore is anti-nociceptive.

The sensory neurons have the innate ability to adapt to stress whereby during disease such as diabetes or peripheral nerve trauma, central sensitisation is induced in the spinal cord. Intrathecal treatment to target spinal cord central sensitisation with VEGF-A_165_b leads to an increase in withdrawal thresholds in naive animals and alleviated neuropathic pain [3]. Alternative splicing by selecting exonal content produces an extended protein family allowing for the expansion of physiological function from a single gene. We have identified that VEGF-A_xxx_a expression (exon8a targeted) is dependent upon serine arginine protein kinase 1 (SRPK1) - serine arginine splice factor (SRSF1) alternative splicing in a number of pathologies as well as chronic pain states [3, 7]. Inhibition of SRPK1 prevents VEGF-A_xxx_a expression and consequently preferentially leads to increased proportion of the anti-nociceptive isoform VEGF-A_xxx_b, thus chronic pain is inhibited [3, 7]. Identifying key alternative splicing events allows for fine tuning the design of pain killing agents by preventing the formation of pro-nociceptive mediators such as VEGF-A_xxx_a though maintaining other essential key isoforms (VEGF-A_xxx_b).

Recent investigations outline that the angiogenic growth factor, VEGF-A, is integral for the modulation of nociception and onset of chronic pain. Understanding these signalling events such as alternative splicing, is key to providing effective and novel analgesia to prevent sensory neuronal damage and chronic pain.
